# ﻿Resurrection of *Passifloraacuminata* DC. and synonymization of *P.tolimana* Harms, *P.gleasonii* Killip, *P.metae* M. Bonilla, C. Aguirre & Caetano (Passifloraceae) following a study of their morphology and ecogeography

**DOI:** 10.3897/phytokeys.201.83316

**Published:** 2022-06-23

**Authors:** Maxime Rome, Geo Coppens d’Eeckenbrugge, John Ocampo Pérez, Mathew Rees

**Affiliations:** 1 Jardin du Lautaret, CNRS, Université Grenoble Alpes, Grenoble, France Université Grenoble Alpes Grenoble France; 2 CIRAD, UMR AGAP Institut, Avenue Agropolis, F- 34398 Montpellier, France UMR AGAP Institut Montpellier France, Metropolitan; 3 UMR AGAP Institut, Univ. Montpellier, CIRAD, INRAE, Institut Agro, F-34398 Montpellier, France Univ. Montpellier Montpellier France, Metropolitan; 4 Universidad Nacional de Colombia, Palmira, Valle del Cauca, Colombia Universidad Nacional de Colombia Palmira Colombia; 5 University of Edinburgh, King’s Building, Edinburgh EH9 3F, UK University of Edinburgh Edinburgh United Kingdom; 6 Royal Botanic Garden Edinburgh,20a, Inverleith Row, Edinburgh, EH3 5LR, UK Royal Botanic Garden Edinburgh Edinburgh United Kingdom

**Keywords:** *Laurifoliae*, subgenus *Passiflora*, taxonomy

## Abstract

Within the very uniform series *Laurifoliae*, *Passifloraacuminata* (treated as a synonym of *P.laurifolia* in the *Flora of China*), *P.tolimana*, *P.gleasonii* and *P.metae* appear particularly similar. A review of their descriptions and the associated specimens confirms their lack of morphological differentiation and leads us to formally resurrect *P.acuminata* and place the three other taxa under its synonymy. This taxonomic move is also supported by a revision of 72 additional geolocalized specimens (for a grand total of 78) and an analysis of their distribution and habitats. In fact, the bioclimatic space corresponding to the specimens previously assigned to *P.acuminata* encompasses that of all specimens previously assigned to the three other taxa under study. The species range covers a wide region, comprising the lower Amazon and the north of its basin, mostly below 200 m, and, to the west, in the upper Amazon, the Orinoco basin, and along the Andean foothills and valleys, from Venezuela to Peru, at elevations between 100 and 2200 m. In the lowlands, the species appears associated with white sand savannas and water courses. A more complete description is proposed for the species, including its unusual fusiform and slightly ribbed fruit. Another rare trait in the series *Laurifoliae* is that the outer corona filaments tend to be longer than the corolla.

## ﻿Introduction

*Passifloraacuminata* DC., *P.tolimana* Harms, *P.gleasonii* Killip, and *P.metae* M. Bonilla, C. Aguirre & C.M. Caetano are very similar taxa that belong to series Laurifoliae Killip ex Cervi, 1997 (subgenus Passiflora, supersection Laurifoliae (Killip ex Cervi) Feuillet & J.M. MacDougal, 2003), which counted 29 species in the classification of MacDougal and Feuillet (2004). The whole series shows a remarkable morphological and ecological uniformity, rendering its taxonomy extremely difficult (Killip 1938). Revising the criteria that delimit it, Rome and Coppens d’Eeckenbrugge (2017) excluded five species from the series *Laurifoliae*. The remaining 24 species are glabrous to pubescent, have stems terete to angular, wingless, sometimes corky on old parts with stipules linear to setaceous, early deciduous. Their leaves are unlobed, oblong-lanceolate, entire to glandular-serrulate, not peltate, and have petioles with 0–2 discoid to oblong sessile glands. Flowers are pendent with two outer series of long campanulate filaments, an involucre of three glandular bracts, free at the base and measuring more than 1 cm long. Since this revision, one more species corresponding to these criteria has been described in Colombia as *P.gustaviana* Ocampo & Molinari, 2017.

*Laurifoliae* species also share habitat preferences, in humid neotropical forests, at low to medium elevations in Central and South America. Furthermore, several species, like *P.nitida* Kunth, *P.riparia* Mart. ex Mast., *P.ambigua* Hemsl., show very extensive distributions (e.g. across the Amazonian basin and/or along the tropical Andes), which favors geographical differentiation and may lead to redundant descriptions in different countries. Rome and Coppens d’Eeckenbrugge (2019) demonstrated such a situation for *P.crenata* Feuillet & Cremers, *P.emiliae* Sacco, *P.fernandezii* L.K. Escobar and *P.pergrandis* Holm-Niels. & Lawesson, and included them in the synonymy of *P.riparia*. Here, we present a similar analysis on *P.acuminata*, *P.tolimana*, *P.gleasonii*, and *P.metae*.

Among these four taxa, the most ancient one is *P.acuminata*, described from Brazil by de Candolle (1828). His very short Latin diagnosis translates as follows: “leaves glabrous, ovate-lanceolate, acuminate, entire; petiole with two glands at the apex; bracts oblong, obtuse, and entire.” De Candolle mentioned a specimen of this species conserved at the National Museum of Natural History of Paris. The latter, without number, had been collected by an anonymous botanist. In *Flora Brasiliensis*, Masters (1872) placed *P.acuminata* as a synonym of *P.laurifolia* L. It was not until 1938 that the species was revived by Killip in *The American species of Passifloraceae*, with a detailed description and citations of several herbarium specimens from Brazil, on which the floral structure can be easily examined. As did Killip, Tillett (2003) distinguished *P.acuminata* from *P.laurifolia* by its radii in two equal series (vs. two unequal series). However, in 2007, this species was again treated as a synonym of *P.laurifolia* in the *Flora of China* (Flora of China Editorial Committee 2007). This rearrangement was not followed, and many authors (e.g. Vanderplank and Zappi 2011, Silva et al. 2013, 2017, Bonilla Morales et al. 2016) still mention it as a distinct species.

**Plate 1.**
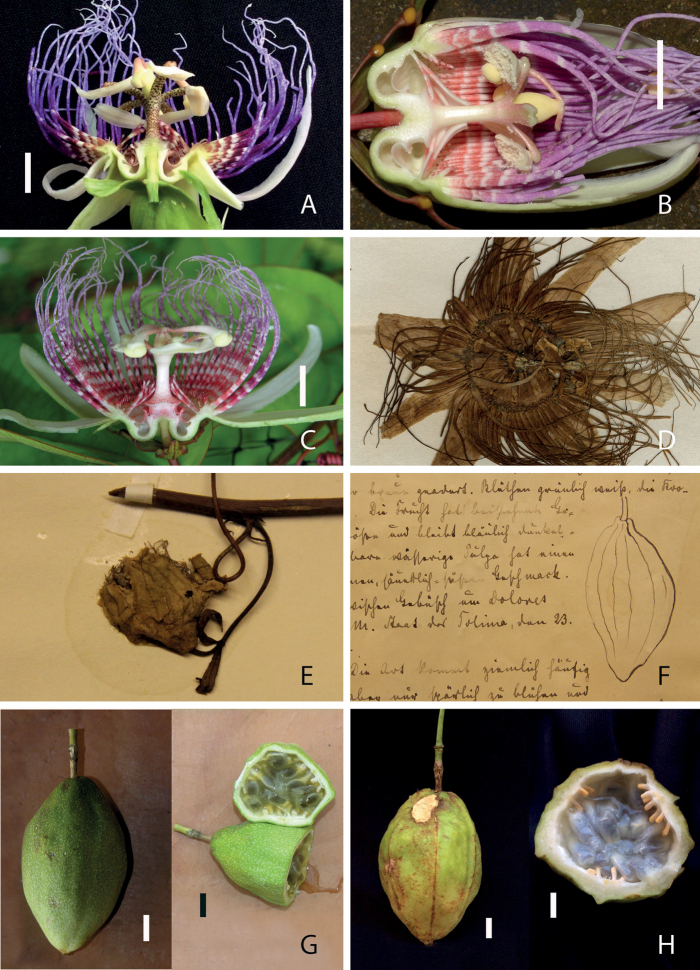
Flowers and fruits of the four taxa under study **A–C** longitudinal sections of flowers of *P.acuminata*, respectively from Santarém, Pará, Brazil (photograph Luis Otávio Adão Teixeira), from Shushufindi, Sucumbíos (initially determined as *P.tolimana*), Ecuador (photograph David Scherberich); and from Guayabones, Mérida, Venezuela (initially determined as *P.gleasonii*, photograph Miguel Molinari **D** dried flower showing the innermost series of filaments, more or less fused, isotype of *P.gleasonii*, Gray herbarium, Harvard **E**, **F** dried flower with ensiform bracts and drawing of fruit, holotype of *P.tolimana*, Kew Garden herbarium (photograph Maxime Rome) **G** fruits of a specimen from Venezuela (initially determined as *P.gleasonii*, photograph Miguel Molinari) **H** fruit of *P.acuminata* from Santa Bárbara, Pará, Brazil (photograph Luis Otavio Adão Teixeira). Scale bar: 1 cm.

In January 1886, in the municipality of Dolores (Tolima, Colombia), at elevations of 1400–1800 m, Lehmann collected a passionflower with greenish white flowers and a corona of purple filaments. He was careful to note that its glossy dark green leaves had a tough texture and, often, brown nervation. The fruit seemed to remain dark green at maturity with a pleasant and sweet taste. Lehmann drew it on the label as a relatively fusiform and ribbed fruit (Plate 1F), not resembling the globular yellow fruits generally observed in series *Laurifoliae*. Finally, he mentioned that the species is quite common but seems to produce fruit only rarely. A few years later, Harms (1894) described meticulously Lehmann’s specimen as *Passifloratolimana*. For the corona, he mentioned that the outermost filaments are slightly longer than the petals, while the innermost filaments are sometimes fused, forming a membrane.

In 1924, Killip described *Passifloragleasonii* from a herbarium specimen collected by J.S. De La Cruz in 1923, on the banks of the Pomeroon River in British Guyana. In the original description, he noted that the species had only three series of filaments, with two outer subequal series, capillary, 4–5 cm long, and an inner series, narrowly linear, 1 mm long. Finally, he mentioned that this species is close to *Passifloranitida* Kunth, but differs from the latter by the outer filaments being narrower and bracts narrowed at both ends, resembling those of *Passifloravitifolia* Kunth.

In 2016, Bonilla Morales et al. described *P.metae* from two very close sites (within 1 km) at Villavicencio in the Meta department of Colombia. Their description and diagnostic key to other Colombian species underline the glabrous ovary (vs. pubescent in *P.tolimana* and *P.gleasonii*); however, most characters remain the same.

In the present article, we compare the descriptions of *P.acuminata* with those of *P.tolimana*, *P.metae* and *P.gleasonii* and study the type specimens and other herbarium specimens cited in their descriptions. Field observations of flowers and fruits corresponding to *P.gleasonii* from Venezuela, *P.acuminata* from Pará (Brazil), and *P.tolimana* from Ecuador, are presented. We thus show that they cannot be distinguished on their morphology. Based on the examination of 78 geolocalized field observations and herbarium specimens, we analyze their distribution and habitat, using Ecological Niche Modeling tools and available label information. Finally, we resurrect formally *P.acuminata*, provide a complete and precise description of it, and place the names of *P.tolimana*, *P.metae* and *P.gleasonii* in its synonymy.

## ﻿Materials and methods

### ﻿Morphological analyses

We first compare the descriptions of *P.acuminata* by Killip (1938) and Tillett (2003), *P.tolimana* by Harms (1894) and Killip (1938), the original description of *P.metae* by Bonilla Morales et al. (2016) and those of *P.gleasonii* by Killip (1924, 1938) and Tillett (2003). This comparison is focused on traits relative to stems, stipules, petiole, leaves, bracts, calyx, corolla, corona (outer and innermost series of filaments), operculum, limen, ovary, and fruit.

A second comparison bears on 15 of the 17 vouchers associated with the above-mentioned descriptions, focusing on the following traits: petiolar gland position, plant pubescence, bract size, and perianth parts, as observable or mentioned on the voucher label. The specimen Ducke 10528, cited in Killip’s description of *P.acuminata*, could not be obtained for examination. The specimen Bonilla 197 (paratype of *P.metae*) had not been deposited at CUVC. No voucher was associated to Tillett’s description of *P.gleasonii*.

We further include our own observations on living material from Ecuador (Sucumbíos), Colombia (Meta, Santander), Venezuela (Mérida, Táchira, Zulia), and Pará (Brazil), other vouchers (newly determined), as well as good and precisely geolocalized photographs from several botanists and/or passionflowers enthusiasts.

#### Examined herbarium specimens

The following list consists of specimens that were previously assigned either to one of the four taxa under study (*P.acuminata*, *P.tolimana*, *P.gleasonii*, and *P.metae*), or to other *Passiflora* species, and now re-assigned to or kept as *P.acuminata* by the authors.

##### Previously assigned to *Passifloraacuminata*

**Brazil**: **Amazonas**: unknown collector s.n. (holotype, P). Lago de Tefé, Manua, 13 Jul 1973, Lleras 16644 (NY). Manaus, along road to Caracaraí, 16 Aug 1986, Croat 62222 (MO). **Pará**: Pará, Ilha de Marajó, Salvaterra, 2 Mar 2012, Costa 534 (MG). 27 Oct–7 Nov 1929, Killip 30272 (US). Obidos, 19–20 Jul 1934, Swallen 5095 (US). Ilha do Mosqueiro, 3–9 Nov 1929, Killip 30572 (US). Santarém, Alter do Chão, between 1998 and 2005, Knowles 1555 (MG). Belém, near Instituto Agronômico do Norte, 28 Oct 1959, Kuhlmann 386 (SPF). Rodovia Belém-Brasília, Km 93, 14 Sept 1959, Kuhlmann 361 (SPF). Santarém, 16 Sept 1999, Cordeiro 4089 (MG). Belém, Parque Estadual do Utinga, 19 Sept 2010, Silva 10 (MG). Belém, Reserva do Mocambo, 23 Nov 2009, Kerpel 8 (MG). Município Almeirim, Mount Dourado, 23 Dec 1986, Pires 1560 (UPCB). South of Instituto Agronômico do Norte, Belém, 25 Nov 1942, Archer 7864 (K). Road from Belém to Viguié, 6 Apr 1961, Aubréville 198 (P). Município de Oriximiná, Rio Trombetas, 22 Apr 2007, Salomão 902 (MG). Belém, Parque Estadual do Utinga, 19 Nov 210, Silva 06 (MG). Belém, on lands of IAN, 20 Jun 1944, Silva 243 (IAN). Santarém, shore of Amazon, 15 Mar 1857, Spruce 760 (K). Belém, Fazenda Murutucú, 22–23 May 1924, Zerny s.n. (W). Porto Trombetas, 26 Apr 1987, Knowles s.n. (INPA). South of Belém near mouth of Rio Guama, 14 Aug 1986, Croat 62142 (MO). Cachoeira Porteira, 17 Jan 1991, Knowles 1696 (INPA). Oriximiná, 23 Jan 2013, Koch 528 (RB). **Roraima**: Amazonas (now Roraima), Caracarai. estrada Manaus, 10 Mar 1978, Silva 4557 (NY). **Colombia: Antioquia**: Municipio San Carlos, Corregimiento Norte del Samaná, 12 Nov 1989, Escobar 8835 (HUA). **Meta**: Municipio de Restrepo, Sector Mirador, 23 Jan 2018, Ocampo 48 (CUVC). **French Guiana**: Monts Tumuc Humac, 30 Aug 1972, Degranville 1430 (CAY). **Guyana: Demarara-Mahaica region**: Along Linden-Soesdyke Highway, 7 Dec 1986, Pipoly 9142 (US). Berbice savanna near Takama Army Base, 14 Aug 1993, Henkel 2520 (NY). **Cuyuni-Mazaruni**; Kako River, 12 May 12 2009, Wurdack 4954 (NY). **Upper Demerara-Berbice region**: Linden-Soesdyke Highway, between Dora and Maibia Creek, 21 Jan 1987, Pipoly 9707 (US).

##### Previously assigned to *Passifloratolimana*

**Colombia: El Valle**: La Cumbre, 2000 meters, 14–19 May 1922, Pennell 5754 (N), Killip 5594 (N), 1 Oct 1922, Killip 11679 (N). **Guaviare**: 1844, Goudot 10 (P). **Santander**: Municipio de Zapatoca, vereda La Cacica, Reserva La Montaña Mágica “El Poleo”, parte alta, 28 Jul 2014, Díaz 829 (MEDEL). **Tolima**: Dolores, 23 Jan 1886, Lehmann 6060 (K). **Ecuador**: **Sucumbíos**: Shushufundi, 4 Dec 2014, Rome 538 (LYJB).

##### Previously assigned to *Passifloragleasonii*

**Brazil: Acre**: Municipality of Rio Branco, road to Xapurí, 24 Oct 1980, Cid 3035 (NY). **Colombia: Guaviare**: Municipality of San José del Guaviare, Inspección de Puerto Arturo, 25 Aug 1995, Cárdenas 6533 (COL). **Guainía**: Maimachi, Serranía del Naquén, 11 Apr 1993, Madriñán 1014 (MO). **Guyana**: Pomeroon district, Pomeroon River, 14 Jan 1923, De La Cruz 2963 (GH). Essequibo River: Moraballi Creek, 14 Sept 1929, Sandwith 254 (K). **Peru**: **Pasco**: Oxapampa, 7 Jul 2003, Werff 18100 (MO). **Venezuela**: **Amazonas**: Department of Río Negro, 9 Mar 1984, Liesner 16501 (MO). **Táchira**: East of Ayarí, 7 Nov 1979, Steyermark 119472 (MO), Cerro de Cuite, along Quebrada La Colorada, 8 Nov 1979, Steyermark 119657 (MO), Cerro of Cuchilla, La Pabellana, 6 Nov 1979, Steyermark 119423 (MO). **Zulia**: 6 km east north-east of Río de Oro, 28 Mar 1982, Liesner 13280 (MO).

##### Previously assigned to *Passiflorametae*

**Colombia**: **Meta**: Municipality of Villavicencio: road Caño Pendejo, 541 m, 23 Dec 2013, (fl), M. Bonilla, J. Mosquera, K. Pulido & A. Cajar 167 (Holotype CUVC!).

##### Previously assigned to *Passifloraambigua*

**Colombia: Santander**: entre Duitama y Virolín, 6 Nov 1979, Escobar 3056 (HUA).

##### Previously assigned to *Passifloralaurifolia*

**Brazil**: **Amazonas**: Manaus; reserva campina - BR174 km 45, Benson 8282 (NY). Igarapé Ipiranga, 4 Jul 1993, Ribeiro 997 (INPA). Estrada para Igarapé do Tinga, 7 Aug 1996, Sothers 883 (INPA). **Pará**: Cachoeira Porteira, 16 Jan 1991, Knowles 1687 (INPA). Mineração Rio do Norte, Porto Trombetas, Mina Saracá, 6 Jun 1999, Miranda 397 (NY). **Colombia: Caquetá**: Orillas del Río Caguán, 12 Apr 1953, Romero-Castañeda 3963 (COL). **Cundinamarca**: Ubala B. Inspección de Policía San Pedro de Jagua, Vereda Soya, 5 Jul 1998, Fernández 16513 (RB). **Meta**: Cordillera de la Macarena, 30 Dec 1950, Idrobo 868 (COL).

##### Previously assigned to *Passifloranitida*

**Brazil: Pará**: Município de Oriximiná, Rio Trombetas, 11 Jul 1980, Cid 1461 (INPA). **Colombia: Antioquia**: San Carlos, corregimiento El Jordán, 29 Sept 1989, Velásquez 241 (HUA). San Carlos, Vereda Patio Bonito, Alto El Cerrón, 17 Feb 1998, Correa 85 (HUA). **Caquetá**: Municipio de Doncello, vereda de Buena Vista, 21 May 2003, Castaño 1649 (COL). **Santander**: Barbosa, bosque en BellaVista, 24 Nov 1940, Pérez Arbeláez 8124 (COL).

##### Previously assigned to *Passiflorariparia*

**Ecuador**: **Morona Santiago**: ca. 32.5 km S of Gualaquiza on road to Zamora, 4 Feb 1984, Knapp 6242 (QCNE). **Napo**: Archidona, 27 Jun 1968, Holm-Nielsen 1040 (AAU).

##### Previously assigned to Passifloraaff.guazumifolia

**Colombia: Boyacá**: Municipio Pajarito, corregimiento de Corinto, 16 Oct 1967, Lozano 935 (COL).

##### Previously assigned to *Passiflora* sp.

**Peru**: **Madre de Dios**: Manu, Aguas Calientes, 13 May 1984, Knapp 6441 (K).

#### Geolocalized data collected by botanists and/or on iNaturalist

##### Passifloraacuminata

**Brazil: Acre**: Porto Acre (D. S. Menezes, https://www.inaturalist.org/observations/41238406). **Amazonas**: BR174, 1.5 km N of Presidente Figueiredo (A. Adair). AM-240, 1 km S of Vila de Balbina (A. Adair). 5 km N of Oriximina (A. Adair). 1 km NE of Obidos along road (A. Adair). Rodovia Eng. Fernando Guilhon, Santarém (A. Adair). **Pará**: Santa Bárbara do Pará (B. Ferreira, https://www.inaturalist.org/observations/34108563 / L. F. Matos (https://www.inaturalist.org/observations/80518762). **Colombia: Antioquia**: Municipio de Remedios, corregimiento La Fragua (J. Restrepo, https://www.flickr.com/photos/22012266@N02/9906490006/in/photostream/). **Meta**: Villavicencio, Reserva Forestal Vanguardia (J. Ocampo P.). **Putumayo**: Puerto Leguízamo (M. Molinari & M. Wettges). **Ecuador: Zamora-Chinchipe**: Yantzatza (A. M. Hualpa Erazo, https://www.inaturalist.org/observations/62721105). **Venezuela: Bolívar**: La Escalera (M. L. Watson, https://www.inaturalist.org/observations/41457638). **Mérida**: La Blanca (M. Molinari). Guayabones (M. Molinari). **Táchira**: Santo Domingo airport (M. Molinari). Colón-San Felix road (M. Molinari).

##### Passifloratolimana

**Colombia: Cundinamarca**: (N. B. Uribe, https://www.inaturalist.org/observations/65638815).

##### Passiflorametae

**Colombia: Cundinamarca**: El Colegio (geolocalization from the photographer, H. Svoboda) https://uk.inaturalist.org/photos/). **Meta**: Buenavista, E of Villavicencio (G. S. Castro, https://www.inaturalist.org/observations/7356149).

### ﻿Analyses of distribution and habitat

After the analyses of descriptions and reference materials showed that the four species under study could not be distinguished on a morphological basis, a geographical database was constituted, including the localizations of 78 herbarium specimens, photographs and observations listed above. These sites were mapped and a global distribution model was developed, using the MAXENT 3.4.1 software and 19 bioclimatic variable layers from the Worldclim 2.1 database at a 2’30” grid resolution (corresponding roughly to 4.4 × 4.6 km at Equator; https://www.worldclim.org/data/worldclim21.html; Fick and Hijmans 2017). MAXENT identifies potential distribution areas based on their similarity in climatic conditions compared to those at the sites where the species has already been observed, hence modeling where conditions are suitable for their development. It infers the probability distribution of maximum entropy (i.e., closest to uniform) subject to the constraint that the expected value of each environmental variable (or its transform and/or interactions) under this estimated distribution matches its empirical average (Phillips et al. 2006). A logistic threshold value equivalent to the 10^th^ percentile training presence was retained to separate climatically favorable areas from marginally fit areas. Thresholds of 33 and 67% training presence were used to discriminate “very good” and “excellent” climates. Furthermore, those bioclimatic covariates that most contributed to the model were extracted for the collection sites, and submitted to a Principal Component Analysis to characterize the bioclimatic spaces corresponding respectively to the previous assignations of specimens.

Three environmental parameters were extracted from specimen labels and/or observed from Google Earth (when highly precise geographical coordinates, from direct GPS measures, were available): proximity of water courses, white sands, and degree of perturbation.

## ﻿Results and discussion

### ﻿Comparative analysis of descriptions

The comparison between the descriptions of *P.acuminata* and the other three taxa is presented in Table 1. The color and pubescence of bracts, and the pubescence of sepals are not given in the table because the descriptions are incomplete regarding these criteria. These traits will be treated in the analysis of herbarium specimens. The series of filaments appear banded with white and purple in all descriptions.

**Table 1.** Comparison of published descriptions of *P.acuminata*, *P.gleasonii*, *P.metae*, and *P.tolimana*.

		*P.acuminata* Killip’s description	*P.acuminata* Tillet’s description	*P.tolimana* Harms’ original description	*P.tolimana* Killip’s description	*P.gleasonii* Killip’s original description	*P.gleasonii* Tillett’s description	*P.metae* original description of Bonilla et al.
stems		terete or subangular	-	rounded to subangular	stem purplish, 5-angled	-	-	terete, striate, glabrous
stipules	length (mm)	about 4	4-8	4-6	4-6	8	8	13
	shape	linear-falcate	narrow-linear, falcate	linear, upper part slightly broader and irregularly glandular-serrate	linear, glandular-serrate toward apex	setaceous	-	linear, falcate, glandular
petiole	length (cm)	about 1	1-1.5	1.1-1.3	1-1.5	up to 2	2	1.7-2
	stipe of glands	-	-	-	-	sessile	sessile or sligthly stipitate	sessile
	gland position	at apex	at apex	at apex	in upper half	below apex	2-5 mm below apex	at apex
leaves	size (cm)	7-14 x 2-5	7-16 x 2-8	10-12 x 3-4	10-20 x 3-10	up to 16 x 9	9-16 x 4.4-9	6-19 × 2.2-5.5
	apex	tapering gradually to an acute apex	-	acute or abruptly acuminate, mucronate	abruptly acuminate, mucronate	abruptly acuminate	obtuse, abruptly acuminate	cuspidate
	base	rounded or acutish	acute, rounded, or subcordate	-	narrowed at base	truncate at base	truncate to shallowly cordate	cuneate
	margin	-	-	entire or very finely denticulate-serrate	entire or remotely and obscurely denticulate	remotely and shallowly glandular-serrulate, or subentire	subentire to shallowly glandular-serrulate-dentate	glandular
	shape	lanceolate or oblong-lanceolate	lanceolate-ovate or oblong-lanceolate	oblong-lanceolate	oblong-lanceolate	oblong	oblong-lanceolate to lanceolate-ovate	elliptic
peduncle	length (cm)	3-4	1-5	2.5-3	up to 3	up to 5	-	3-3.5
bracts	size (mm)	25-40 x 10-15	15-40 x 5-15	-	25-35 x 20	about 20 x 4-5	20-35 x 4-14	15-20 × 4.5-5
	shape	obtuse at apex	narrowly elliptic to oblong	spatulate-obovate to oblong with an acute apex	ovate	oblong-elliptic, cuspidate, acuminate	oblong-elliptic, base narrowed, apex rounded, abruptly cuspidate-acuminate	ensiform
	margin	-	entire or glandular	crenate-serrate with 2-3 glands	glandular serrate	glandular-serrate at apex	basal half with large marginal glands, finely serrate	glandular
sepals	size (mm)	20-25 x 6-7	20-30 x 6-10	about 20	15-20 x 7	30-35 x 10	30-35 x 10	30-32 x 9-10
	shape	lanceolate	narrow lanceolate	oblong	oblong	lanceolate, obtuse	lanceolate, obtuse	lanceolate, round at apex
	color	-	whitish green with purple spots	-	greenish white without, light violet within	-	-	adaxially green, abaxially white
	sub-apical awn	yes	yes	yes	yes	no	yes	yes
petals	size (mm)	about 15 mm long	about 15 mm long	similar to sepals or narrower	similar to sepals, sligthly narrower	20 x 5	20 x 5	30-32 × 7-8
	color	-	white or lavender	-	light violet	-	-	white
series of filaments	number of series	5	5 or 6	-	4 or 5	3	3	5
	outer series length (mm)	30-40	25-40	slightly longer than petals	subequal; slighlty exceeding petals	40-50	40-51	38-40
	inner series	2 series with few filaments, setaceous, barely 1 mm long, innermost filaments subulate, 5-6 mm	3 or 4 series, the 2 or 3 outer few, setaceous, less than 1-2 mm, innermost subulate, 5-7 mm	shorter than outer series, the innest sometimes fused	inner filaments much shorter, united at base	one series linear, 1 mm	linear, 1-2 mm	3d series 2–3 mm, 4th series 0.8–1 mm, innermost series 7.2–7.5 mm, inclined towards androgynophore
oper-culum	length (mm)	4-5	4-5	-	-	7-8	7-8	3
	shape	membranous, slithtly incurved, minutely fimbriate	incurved, fimbriate	membranous, inflexed at base, margin erect, short-toothed	membranous, inflexed at base, margin erect, short-toothed	membranous, cleft to the base into linear segments nearly 3 mm wide	cleft to base in segments to 3 mm wide	membranous, strongly incurved, margin fimbriate, white at base, red at apex
limen		tubular, closely surrounding base of gynophore	-	thick base with two rings, the first above base of receptacle, the second 1.5 mm from the first	annular, closely surrounding the base of the gynophore, about 12 mm long, bearing a thickened, annular process about 1.5 mm above its base	borne close to base of gynophore, barely 2 mm high, denticulate	-	6 mm long
ovary		ovoid, minutely puberulent	ovoid, minutely puberulent	tomentose, elliptic, about 5 mm long	ellipsoidal, short-tomentose	ovoid, finely ferruginous-tomentullous	ovoid, finely ferruginous-velutinous	ovary 5–6 × 3 mm, ellipsoid, glabrous, yellowish green
fruit		-	-	-	-	-	globose to ovoid, 7 x 3.5 cm	unknown

As *P.tolimana* and *P.gleasonii* descriptions were based on herbarium materials, they lack information on perianth color. In the case of *P.tolimana*, the description underestimates the perianth size and lacks the size of the bracts and the operculum.

As shown in Table 1, the comparison of the different descriptions allows no clear distinctions that could justify the consideration of different species. Stems are rounded to angulate, which is the commonest situation in passion flowers. Stipules appear to be short, as compared to the common size in *Laurifoliae*, which is likely related to their observation on dry materials, and slightly longer for *P.metae*, but the information on stipule shape and width or with the scale given in the drawing is very inconsistent for this taxon, hampering any particular conclusion. Petioles are short too, under 2 cm, and their glands are placed close to apex, with the relative exception of Killip’s description of *P.tolimana*. For leaves and bracts, their variation in size, shape and distribution of marginal glands is negligible, falling well within the range of variation observed in other widespread species of series *Laurifoliae* (e.g. *P.riparia*; Rome and Coppens d’Eeckenbrugge 2019).

Similarly, taking into account the effect of desiccation on herbarium descriptions, no taxa show a distinct range of variation for sepals, petals, and corona filaments. Killip’s description of *P.gleasonii* is unique in mentioning no awn on sepals, which would be exceptional for the whole subgenus Passiflora; and it is contradicted by Tillet’s description. In fact, minute awns can be seen on sepals of the isotypes conserved at the Gray herbarium and the Missouri Botanical Garden (897955). The respective lengths given for the corona elements clearly indicate that the two outer series of filaments are longer than sepals and petals, an observation that was reported only in Harm’s description of *P.tolimana*. The description of the innermost series of filaments, intermediate in size, is not explicit in the descriptions of Harms and Killip, however these authors mention their possible fusion at base, which implies that they are not as short as, and denser than, those intermediate series with few filaments 1–2 mm long. Indeed, the analysis of the *P.gleasonii* holotype shows that the operculum described by Killip is a fourth series of striped filaments, more or less fused. Apart from this confusion in Killip’s description, no significant variation has been reported for the operculum and limen. The mention of a 6 mm-long limen by Bonilla Morales et al. (2016) is not supported by the longitudinal flower section drawn and photographed in their paper. In fact, the limen is reduced to a ring widening the trochlea, as in the description of Tillett (2003). Finally, the only potentially significant difference is the report of a glabrous ovary for *P.metae* in the last row of Table 1.

### ﻿Analysis of specimens associated to the descriptions

Table 2 presents the 15 reference specimens cited in the descriptions of *P.acuminata*, *P.gleasonii*, *P.tolimana* and *P.metae* that could be examined. Six of them were sterile or in buds.

**Table 2.** Comparison of herbarium specimens cited in the descriptions of *P.acuminata*, *P.tolimana*, *P.gleasonii* and *P.metae*.

Specimen, Institution	Origin	Petiolar gland position	Bracts	Pubescence	Outer series of filaments	4^th^ series almost perpendicular to the androgynophore, closing the nectary chamber	Hypanthium	Limen	Observations
De Candolle 1828, description of *P.acuminata*									
**Unknown collector s.n., P**	Brazil	apex	2.8 × 1 cm, oblong, margin glandular	bracts	two equal filiform, longer than perianth	–	–	–	Flower too damaged to examine the ovary and the inner series
Killip 1938, description of *P.acuminata*									
**Swallen 5095, US**	Pará (Brazil)	below apex	3 × 2 cm, ovate, margin glandular	ovary	two equal filiform, longer than perianth	yes	reduced to the nectary chamber	reduced to a ring on the trochlea	–
**Spruce 760, K**	Pará (Brazil)	apex	4.2 × 3.9 cm, ovate, margin glandular	ovary	two equal filiform, longer than perianth	yes, filaments more or less fused	reduced to the nectary chamber	reduced to a ring on the trochlea	–
**Burchell 9504, K**	Pará (Brazil)	apex	3 × 1.4 cm, ovate, margin glandular	–	two equal filiform, longer than perianth	yes	reduced to the nectary chamber	reduced to a ring on the trochlea	–
**Burchell 9988, K**	Pará (Brazil)	apex	absent	–	–	–	–	–	sterile specimen
**Killip 30272, US**	Pará (Brazil)	apex	–	not examined	–	–	–	–	specimen without flowers
**Killip 30572, US**	Pará (Brazil)	apex	2.5 × 0.8 cm, lanceolate, margin glandular	not examined	–	–	–	–	specimen without flowers
**Hoffmannsegg, BR**	Pará (Brazil)	apex	more than 3 × 1 cm, lanceolate	–	–	–	–	–	wrinkled bracts, no flowers
Harms 1894, description of *P.tolimana*									
**Lehmann 6060 (Type), K, B, F**	Colombia	apex	11 × 3 mm, ensiform to elliptic, glandular	bracts, ovary	two equal filiform, longer than perianth	yes	reduced to the nectary chamber	reduced to a ring on the trochlea	–
Killip 1938, description of *P.tolimana*									
**Pennell & Killip 5754, US**	Colombia	just above middle	–	–	–	–	–	–	P.cf.ambigua, sterile specimen
**Killip 5594, US**	Colombia	just above middle	–	–	–	–	–	–	P.cf.ambigua, sterile specimen
**Killip 11679, US**	Colombia	at middle	4.5 × 2.5 cm, oblong,	not observable	not observable	not observable	–	not observable	*P.ambigua*
Killip 1924 & 1938, descriptions of *P.gleasonii*									
**De La Cruz 2963 (Type), US, CM, GH, MO**	Guyana	below apex	2 × 0.7 cm, elliptic–lanceolate, margin glandular	bracts – ovary	two equal filiform, longer than perianth	yes, filaments more or less fused	reduced to the nectary chamber	reduced to a ring on the trochlea	–
**Sandwith 254, K**	Guyana	below apex	elliptic, margin glandular	bracts – ovary	two equal filiform, longer than perianth	yes	reduced to the nectary chamber	reduced to a ring on the trochlea	–
Bonilla & al. 2016, description of *P.metae*									
**Bonilla 167, CUVC**	Colombia	apex	–	–	two equal, filiform	yes	–	reduced to a ring on the trochlea	damaged specimen

The type of *P.acuminata* shows a very degraded flower. Thus, while we perceive the presence of two equal outer series of filiform filaments, as well as pubescent bracts with glands, it is impossible to observe the pubescence of the ovary and the presence of the inner series of filaments. From the seven specimens associated to *P.acuminata* in Killip’s description, only Swallen 5095, Spruce 760, and Burchell 9504 exhibit a set of traits sufficient to allow full confidence in their determination, given the common presence of *P.laurifolia*, also having two glands at the apex of the petiole, in the Pará state of Brazil.

Among the four specimens associated with Killip’s descriptions of *P.tolimana*, only the type Lehmann 6060 is representative. The three other specimens, Pennel 5754, Killip 5594, and Killip 11679, present no positive criteria to confirm their identification (and the flower buds on the latter could not be dissected). On the contrary, they present glands on the middle of the petiole, which indicates that they belong to *P.ambigua*, now known from the Cumbre region where these samples were collected. This confusion explains why Killip’s description diverges from the original one on the position of the petiolar glands.

The description of *P.metae* cites the holotype and isotypes Bonilla et al. 187 (deposited at CUVC and at FAUC) and the paratype Bonilla et al. 197 (deposited at CUVC). However, the only specimen available is present at CUVC, where it is given as the holotype and referenced as Bonilla et al. 167. Its flower is in very poor condition and the androgynophore has been broken, which prevents from verification of the glabrousness of the ovary, i.e., the only potentially distinctive trait of this taxon.

Except for the three specimens associated to Killip’s description of *P.tolimana*, which are likely representatives of *P.ambigua*, all the examined specimens present leaves with two glands at the petiole apex (or just below), bracts with glandular margins, variable pubescence on the ovary and sometimes on other floral parts as well (bracts, peduncle and calyx), a hypanthium reduced to the length of the nectary chamber, two equal outer series of filaments, longer than the perianth, a fourth series of filaments almost perpendicular to the androgynophore closing the nectary chamber, and a limen reduced to a ring at the base of the trochlea. On the specimens De La Cruz 2963 and Spruce 760, the fourth, innermost, series shows filaments more or less fused together.

### ﻿Observations on additional specimens

In total, we have analyzed 55 herbarium specimens and 23 photographed specimens of *P.acuminata*, including herbarium specimens that had been previously determined as representatives of *P.laurifolia* (eight cases), *P.nitida* (five cases), *P.riparia* (two cases), *P.ambigua* (one case), P.aff.guazumifolia Jussieu (one case), and two undetermined specimens. These redeterminations were mostly based on hypanthium length (reduced to the nectary chamber in *P.acuminata*), the inner series of filaments, about 1 cm, absent in *P.laurifolia*, *P.riparia* and *P.ambigua*, or parallel to the androgynophore in *P.nitida* (vs. perpendicular in *P.acuminata*), and the position of petiolar nectaries (in apical position vs. median position in *P.ambigua* and *P.riparia*).

Plate 1 presents the high similarity observed for flower and fruit traits, even among materials collected at considerable distances. Photographs A, B, C, D, respectively from Pará (Brazil), Sucumbíos (Ecuador), Mérida (Venezuela) and Guyana, show the same floral structure, which can also be clearly observed on the photograph of the holotype of *P.metae* presented by Bonilla Morales et al. (2016). There are two equal outer series of filaments, longer than the perianth. The inner series are aborted while the innermost series, about 1 cm long, close the entrance of the nectary chamber. The hypanthium is short and reduced to the nectary chamber. The androgynophore is twice wider at its base than above. It can be more or less punctuated with purplish red spots, as can the stamen filaments.

As mentioned by Lehmann for *P.tolimana* (Killip 1938), fruits are rare, however we could gather observations from Colombia, Brazil and Venezuela (Díaz 829; photographs F, G, H), showing strikingly similar fruits, elliptic to fusiform, slightly ribbed, yellowish green, with a soft epicarp and very thin mesocarp, subhexagonal in cross sectional view. This convergence on an exceptional trait provides further support to the synonymization of the four taxa.

More variation can be observed in bracts and perianth color. Thus, we find various forms, with limited geographic consistency: long and broad bracts north of the Amazon (from the Brazilian states of Pará and Amazonas to the south of Venezuela), small and elliptical bracts in the Brazilian Amazonas, Guyana, Colombia and Ecuador, ensiform to short and wide bracts in Colombia and Venezuela. This diversity, coupled with the under-sampling and poor knowledge of this species at the time of the original descriptions, partly explains the multiplication of taxa. In their study of *P.riparia* and its synonyms, Rome and Coppens d’Eeckenbrugge (2019) also noted the relatively wide intraspecific variability of bract size and shape.

The color of the perianth is predominantly white. However, we found two specimens from Venezuela and Colombia with purplish petals and sepals and exhibiting all of the other characteristics of *P.acuminata* (Liesner 16501 and J. Restrepo’s photograph). There is also variation in the darkest color of the outer series of filaments, which can range from pink to dark purple, a variation that is also observed within *P.nitida*.

As observed in the specimens associated to the descriptions, there is some variation in the pubescence of the corolla elements and the ovary. While we could not verify the presence of a glabrous ovary on the type of *P.metae*, we could observe a weak pubescence on the ovary of specimens observed very close to the *locus classicus* in the Colombian department of Meta.

In contrast with several other species in the series *Laurifoliae* (e.g. *P.riparia* and *P.ambigua*), we have not observed flowers gathered in pseudoracemes in the analyzed materials.

### ﻿Distribution and ecology

As shown by the global list of examined specimens, *P.acuminata* has so far been documented essentially in Brazil, the country where it was first described. It appears particularly common around Belém, in northeastern Pará and northern Maranhão, but there may be a collecting bias related to the proximity of the Goeldi Museum located at Belém. It had not been collected south of the lower Amazon River (which is consistent with field observation by A. Adair, pers. com.). Elsewhere, in Colombia, Venezuela, and the Brazilian state of Acre, specimens identified as *P.acuminata* correspond to determinations of recent photographs in the framework of the present study, except for Liesner 13280, collected in the Venezuelan state of Zulia, close to Colombia.

A similar situation is observed for *P.tolimana*, described in the eastern Andes of Colombia, with observations and collections relatively concentrated around this same Tolima region and, to the south, in the Andes of Ecuador (including Holm-Nielsen1040 and Knapp 6242, as redetermined by Rome and Coppens d’Eeckenbrugge 2019), and Peru.

The name of *P.gleasonii* dispersed much further. Indeed, Killip described it from Guyanese specimens, however, all later collections are from Andean foothills, in Colombia and Peru, from Amazonian lowlands, in the southern Orinoco region of Venezuela and Colombia, and western Brazil (upper Amazon and Acre).

The case of *P.metae* is particular as the only geolocalized specimen authenticated by Bonilla Morales is the one photographed by Steven Castro very close to the locus classicus, and very close to specimens of *P.acuminata* with a pubescent ovary.

**Figure 1.**
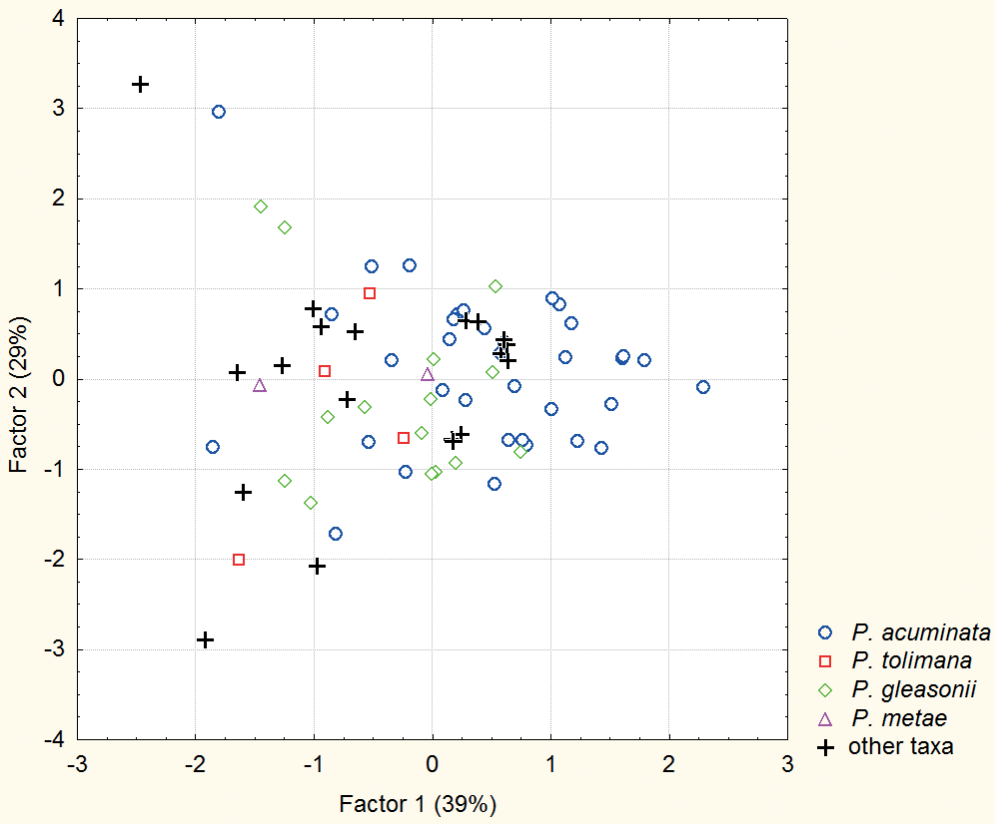
Principal plane of the Principal Component Analysis on the eight bioclimatic variables that most contributed to the *P.acuminata* distribution model built from 78 geolocalized specimens. Factors 1 and 2 account for 39 and 29% of total variance respectively. The symbols indicate the initial assignation of specimens.

The analysis of the MAXENT model derived from our global dataset (all geolocalized specimens irrespective of their previous assignment) shows that the most determinant bioclimatic variables are the temperature diurnal and annual ranges, the maximum temperature of the warmest month, the mean temperature of the warmest quarter, the precipitation seasonality, with the precipitations of the driest month and those of the driest and coldest quarters. These variables were thence selected for the Principal Component Analysis. The principal plane, accounting for two thirds of the total variance, allowed a fairly good representation of the bioclimatic space of the geolocalized specimens (Fig. 1). The first and main observation is the absence of any appreciable differentiation in climatic adaptation between the four taxa under revision. Indeed, the climatic envelope occupied by specimens that had been assigned to *P.acuminata* encompasses that of all the other specimens. Noticeably, the small group of ten specimens showing a score above 1 on the first axis (extreme right of the plane) were all collected close to Belém (seven cases) and Santarém (three cases).

**Figure 2.**
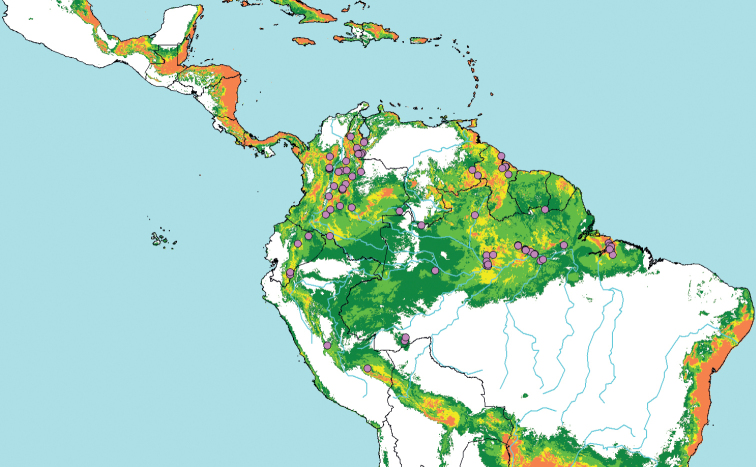
Potential distribution model for *P.acuminata*; derived from the 78 geolocalized specimens of the present study (pink circles) and MAXENT bioclimatic modeling. Background colors indicate climate suitability: marginal (dark green), favorable (light green; above 10% training omission threshold), very favorable (yellow; above 33% omission threshold); excellent (orange; above 67% omission threshold).

Fig. 2 presents the geographic space corresponding to this climatic envelope, i.e., the potential distribution of *P.acuminata* across South America and Central America. This wide distribution appears to be split between two major regions: to the east, from the Guianas to the lower Amazon River, up to the Rio Negro and southeastern Venezuela, favorable climates are mostly found at low elevations, well under 200 m (except for de Granville 1340, Wurdack 4954, and Watson’s observation, from French Guiana, Guyana and Venezuela, at elevations around 500 m); to the west, in the upper Orinoco region and along the Andean foothills and valleys, from western Venezuela to Peru, *P.acuminata* has mostly been reported at elevations comprised between 200 and 2200 m. However, it is also present below 200 m, in Andean valleys and low Orinoquian and Amazonian regions of Venezuela and Colombia. These major, eastern and western, regions are linked by extensive lowland areas where climates are mostly classified as marginal to the species and its presence appears sporadic. However, some of these areas are of difficult access and have been poorly explored (e.g. basins of the Javari River in western Brazil and the upper Amazon River in Peru, except for the vicinity of Iquitos).

On the other hand, several areas presenting favorable to excellent climates according to the MAXENT model, are not supported by effective observations of the species. In the eastern region, the absence of the species south of the lower Amazon, as observed by A. Adair (pers. com.) is not precisely explained by the model, as the climate appears favorable there. More strikingly, while several highly favorable areas are predicted along the shores of Suriname and French Guiana, the presence of *P.acuminata* has never been confirmed in northern French Guiana despite extensive collecting activity (Rome and Coppens d’Eeckenbrugge 2016, 2020). In the western region, the distribution of specimens mostly correlates with the MAXENT model results for areas along the northern Andes (Colombia and Ecuador). The relative rarity of observations in the climatically favorable upper Orinoco can be easily explained by the lack of botanical exploration in this part of Colombia (Ocampo Pérez et al. 2010). But such an explanation does not hold for the absence of observations in the Colombian Western Cordillera as well as in the Colombian, Ecuadorian and Panamanian Chocó, and further north into Central America, all contiguous areas whose climates appear highly favorable for *P.acuminata*. Indeed, *P.nitida* and *P.ambigua* have been repeatedly collected there, but no *P.acuminata*.

The MAXENT bioclimatic model also points to very favorable areas along the Central Andes, southward to Bolivia and southern Brazil, but they are not met by any observations beyond Peru.

The lack of observations of *P.acuminata* in several extensive areas that combine highly favorable climates and a relatively good level of botanical prospection suggests that the species distribution is significantly constrained by non-climatic factors, justifying further ecological analyses. Before such studies are carried out, our MAXENT distribution model can be considered as a guide for collecting more data.

The ecological information associated with herbarium specimens and the precise geolocalization associated with iNaturalist photograph databases allow inquiring on habitat parameters at a smaller scale, either from label information or from available geographic information (e.g. aerial views).

As for *P.riparia* (Rome and Coppens d’Eeckenbrugge 2019) and *P.nitida*, *P.acuminata* appears associated to riparian habitats. Out of our 78 records, 69 provide information on humidity, with 48 positive cases (close proximity to streams, lakes, ponds, or temporarily flooded areas). Another frequent element of habitat descriptions is the mention of sandy soils, mostly white sand savanna or “campina” habitat (31 cases), particularly in Amazonian and Orinoquian lowlands. Although not signaled in the specimen label, these white sands are very common in the Guyanese region of the *locus classicus* of *P.gleasonii*. In contrast, only one case of clay soil and one of rocky soil are signaled. A rocky soil is also likely for Idrobo 868, collected on the Cordillera Macarena slopes in Colombia. Finally, 51 records point to disturbed vegetations, in contrast with only eleven simply mentioning a forest habitat. The latter is not surprising for a liana, benefitting from clearings in the forest, and easier to observe and collect along roadsides and in open habitats.

This first ecogeographical approach has provided no evidence of differential climatic adaptation among specimens previously assigned to *P.acuminata*, *P.tolimana*, *P.gleasonii* or *P.metae*. The available herbarium information points to a common association with riparian habitats and/or white sand soils, particularly in lowland areas. Further studies, involving soil information, and more collections, are needed to identify non-climatic factors limiting the presence of *P.acuminata* in a few climatically favorable areas.

### ﻿Taxonomic treatment

**Plate 2.**
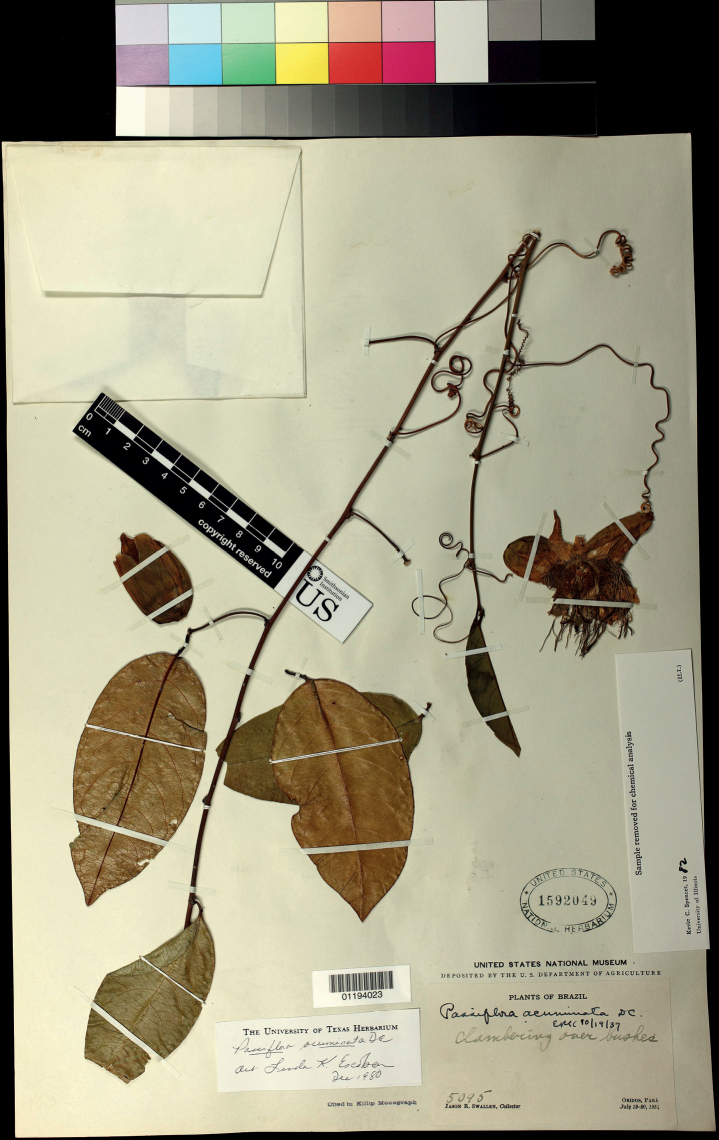
Specimen Swallen 5095 [US01194023], designated as epitype of *Passifloraacuminata* (copyright US National Herbarium).

All previous observations confirm that it is not possible to differentiate several species among the four taxa under study on morphological and/or ecological bases. Thus, it is necessary not only to resurrect *P.acuminata* but also to place the other species as synonyms of the latter. Furthermore, as the type of this species is not sufficiently explanatory, we propose an epitype, Swallen 5095 (Plate 2), presenting all the elements necessary for the morphological understanding of the species, following the suggestion of the international code of nomenclature (Turland et al. 2018).

#### ﻿ Passiflora acuminata

Taxon classificationPlantaeMalpighialesPassifloraceae

DC., Prodr. [A. P. de Candolle] 3: 328. 1828.

26C1321A-5287-55B3-B95B-C1DF9C10FD54

Passiflora tolimana Harms, Bot. Jahrb. Syst. 18 (Beibl. 46): 9. 1894, syn. nov. Type: Colombia, Tolima: Dolores, 23 January 1886, *F.C. Lehmann 6060* (holotype: K! [K000323465]; isotypes: B![B16561], F![V0066827F])
Passiflora gleasonii Killip, J. Wash. Acad. Sci. 14: 112–113. 1924, syn. nov. Type: Guyana: Distr. Pomeroon. Along the Pomeroon River, 14–20 Jan 1923, *J.S. de la Cruz 296*3 (holotype: US! [US1123194]; isotypes: CM! [CM164669], GH! [GH3043413], MO! [MO3043413]).
Passiflora metae M. Bonilla, C. Aguirre & C. Caetano, Phytotaxa 267(2): 130, f. 1–5. 2016, syn. nov. Type: Colombia, Meta: Villavicencio Municipality: road Caño Pendejo, 541 m, 23 December 2013, *M. Bonilla*, *J. Mosquera*, *K. Pulido & A. Cajar 167* (holotype: CUVC! [CUVC067410]).

##### Type.

Brazil. Anonymous, s.n. (holotype, P! [P00605761]). Brazil, Pará, Obidos, 19–20 July 1934, *Swallen 5095* (epitype: US! [US1592049]) designated here.

##### Description.

Woody liana. Stems rounded to angular, glabrous and green. Tendrils conical, glabrous. Stipules linear, upper part slightly broader and irregularly glandular-serrate, glabrous, 4–18 mm long, about 1 mm wide, deciduous. Petiole 1–2 cm, green to reddish green, slightly canaliculate on the upper part, glabrous, with two sessile and ovate glands (young glands pyramidal) below the apex or at the apex of petiole. Leaves simple, 6–19 × 2.2–9 cm, glabrous throughout, green to dark green, upper surface lustrous, ovate lanceolate to elliptic, cuneate to cordate at base, mucronate and acuminate; margin entire to glandular-serrulate; nerves often reddish. Peduncle terete, green to reddish green, glabrous to slightly pubescent, about 1–2 mm in diameter, 25–50 mm long; pedicel 4–8 mm long. Bracts persistent (until complete ripeness of fruit), ovate to narrow elliptic, apex rounded to acuminate, base cuneate to rounded, glabrous to slightly pubescent, green to dark red, concave, 15–40 mm long, 4–15 mm wide, with a margin glandular to glandular-serrulate. Flowers, axillary, pendulous, about 3 cm long (from the nectary chamber to the ovary apex), solitary. Hypanthium (including the nectary chamber) slightly pubescent, green with red dots outside and white inside, about 5 mm long, with a diameter of about 10 mm at the sepal base. Sepals glabrous to slightly pubescent, oblong, 20–32 mm long, 6–10 mm wide, white (rarely lavender), slightly keel-shaped in distal half with a short to medium awn (1–5 mm long). Petals glabrous, oblong, 15–32 mm long, 5–8 mm wide, white (rarely lavender). Corona of filaments in five series, banded with white and red to purple or dark violet; two major outer series equal 38–51 mm long, the third series 2–3 mm long, the fourth series 0.8–1 mm long, the innermost series, about 10 mm long, with filaments sometimes fused at base, almost perpendicular to the other series, closing the hypanthium entrance. Staminal filaments 9–10 mm long, greenish white with red dots. Ovary pubescent, 5–6 × 3 mm, ellipsoid, yellowish green; three styles (their base can be pubescent), white with red dots, 14–15 mm long, stigmas light yellow. Androgynophore glabrous, greenish white, slightly to densely dotted with red, 20–23 mm long with an enlarged base, with two bulges about 10 mm in diameter. Limen annular, less than 1 mm long. Operculum membranous, translucid-whitish, 3–8 mm long, inflexed at base, the margin erect, short-toothed. Fruit ellipsoid with conical apex to fusiform, pubescent, 6–9 cm long, 3.6–6.5 cm in diameter, triangular to hexagonal in transversal section, slightly ribbed, pericarp 6–10 mm thick; unripe fruit green with minute white dots; ripe fruit yellowish green, minutely dotted, with a sweet translucent pulp. Seeds obovoid, flat, heart-shaped, about 10 mm long.

## ﻿Conclusion

Following our morphological and ecogeographic analyses, the name of *P.acuminata* is resurrected and *P.metae*, *P.gleasonii*, and *P.tolimana* are placed as synonyms of this taxon, which reduces the current number of species belonging to series *Laurifoliae* to 18. Thus, this species is unique by its combination of characters: two nectar glands at the apex of petiole, two equal outer series of filaments, longer than petals and sepals, a hypanthium reduced to the nectar chamber, an innermost series of filaments closing the nectar chamber, a slightly pubescent ovary, and an elliptical to fusiform fruit, triangular to hexagonal in transverse section, with a thin pericarp. In the series *Laurifoliae*, its floral structure is similar to that of *P.kapiriensis* Rome & Coppens; however the latter has wider leaves, rounded fruits and two glands at the middle of the petiole.

Like several other species in the series, such as *P.laurifolia*, *P.nitida* or *P.riparia*, *P.acuminata* is widely distributed in the basins of the Amazon and the Orinoco and in Andean hillsides, and these four species probably show an appreciable level of sympatry. In the Andes, it might also share habitat with *P.ambigua* (Rome and Coppens d’Eeckenbrugge 2019). Further revision in the series may confirm that *Laurifoliae* species with a narrow distribution are less frequent than previously thought; this renews our interest in the mechanisms that subtend the evolution of species that are so similar in their morphology and ecology, and thence sympatric in wide areas within the Neotropics.

## Supplementary Material

XML Treatment for Passiflora acuminata

## ﻿Index to numbered collections

Archer, W.A. 7864 (acuminata)

Aubréville, A. 198 (acuminata)

Bonilla, M. 167 (acuminata)

Cardenas, D. 6533 (acuminata)

Castaño, N. 1649 (acuminata)

Cid, C.A. 1461, 3035 (acuminata)

Cordeiro, M.R. 4089 (acuminata)

Correa, E. 85 (acuminata)

Costa, R. 534 (acuminata)

Croat, T. 62142, 62222 (acuminata)

De La Cruz, J. S. 2963 (acuminata)

Degranville, J. J. 1430 (acuminata)

Díaz-R., D. 829 (acuminata)

Escobar, L.A. 3056, 8835 (acuminata)

Fernández, J.L. 16513 (acuminata)

Goudot, J. 10 (acuminata)

Henkel, T.W. 2520 (acuminata)

Holm-Nielsen, L. 1040 (acuminata)

Idrobo, J.M. 868 (acuminata)

Kerpel, S.M. 8 (acuminata)

Killip, E.P. Killip 5594, 11679 (cf. ambigua), 30272, 30572 (acuminata)

Knapp, S. 1555, 6242, 6441 (acuminata)

Knowles s.n., 1687, 1696 (acuminata)

Koch, A.K. 528 (acuminata)

Kulhmann, J.G. 361, 386 (acuminata)

Lehmann, F.C. 6060 (acuminata)

Liesner, R. 13280, 16501 (acuminata)

Lleras, E. 16644 (acuminata)

Lozano, G. 935 (acuminata)

Madriñan, S. 1014 (acuminata)

Miranda, I.P. (acuminata)

Ocampo, J.A. 48 (acuminata)

Pennell, F.W. 5754 (cf. ambigua)

Pérez Arbeláez, E. 8124 (acuminata)

Pipoly, J.J. 9142, 9707 (acuminata)

Pires, M.J. 1560 (acuminata)

Ribeiro, J.E.L. S. 997 (acuminata)

Rome, M. 538 (acuminata)

Romero-Castañeda, R. 3963 (acuminata)

Salomão, R. de P. 902 (acuminata)

Sandwith, N.Y. 254 (acuminata)

Silva, E.O. 06, 10, 243, 4557 (acuminata)

Sothers, C.A. 883 (acuminata)

Spruce, R. 760 (acuminata)

Steyermark, J.A. 119423, 119472, 119657 (acuminata)

Swallen, J.R. 5095 (acuminata)

unknown collector s.n. (acuminata)

Velásquez, M.P. 241 (acuminata)

Werff (van der), H. 18100 (acuminata)

Wurdack, K.J. 4954 (acuminata)

Zerny, H. s.n. (acuminata)
